# Preparation of a novel polyacrylic acid and chitosan interpenetrating network hydrogel for removal of U(vi) from aqueous solutions

**DOI:** 10.1039/c7ra13065a

**Published:** 2018-04-03

**Authors:** Jiarui He, Fuliang Sun, Fuhao Han, Junjie Gu, Minrui Ou, Wenkai Xu, Xiaoping Xu

**Affiliations:** College of Chemistry, Fuzhou University Fuzhou 350108 China xu@fzu.edu.cn

## Abstract

A clean and simple method has been developed for preparation of interpenetrating polymer networks using polyacrylic acid (PAA) and chitosan (CS) for extraction of uranium from polluted water. The peak of Fourier transform infrared spectroscopy (FTIR) occurred at 928 cm^−1^ indicating combination of uranium and PAA/CS. The energy dispersive X-ray (EDX) and the scanning electron microscope (SEM) studies illustrated the formation of a crosslinking structure and excellent binding ability of uranium on PAA/CS. The maximum adsorption capacity was 289.6 mg g^−1^ calculated using the equation of the Langmuir model. The adsorption capacity reached a plateau at pH 4 and the sorption process fits the pseudo-second-order model well. The PAA/CS composite has stability of reuse, with the adsorbent capacity decreasing slowly with increasing usage frequency. The experimental results confirm that the PAA/CS hydrogel could be a novel alternative for highly efficient removal of uranium from wastewater.

## Abstract 2

A clean and simple method has been developed for preparation of interpenetrating polymer networks using polyacrylic acid (PAA) and chitosan (CS) for extraction of uranium from polluted water.

## Graphical abstract

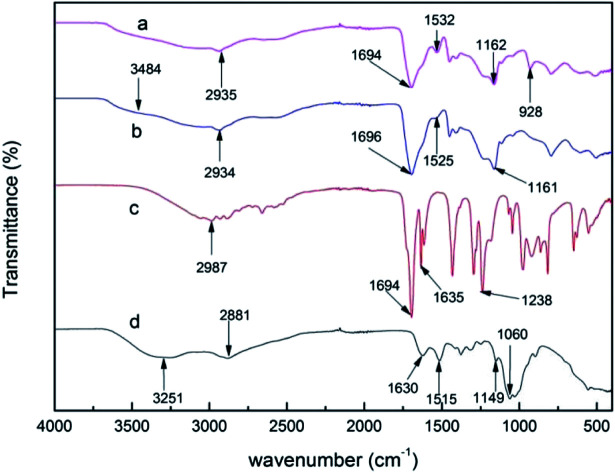

## 1. Introduction

Conventional energy sources from the degradation of coal and petroleum cause serious environmental and human health problems. The high speed development of nuclear energy as an alternative means that various radionuclides are being released into the environment from the legacy of mining, nuclear reactors, and activities of nuclear weapons.^1^ The uranium ion is one of the most common contaminating ions, with high radiation and chemical toxicity. Therefore, focus in recent years has been on how to reduce the uranium ion content in drinking water. Although various methods are available to reduce uptake of radioactive elements in various fields, efficient techniques are still required.

There are many physical and chemical methods for removal of uranium from wastewater including instance ion-exchange processes,^2^ solvent extraction,^3^ surface complexation, and adsorption.^4–6^ Adsorption has been used by many plants and enterprises because of its excellent performance, easy operation, low consumption, high efficiency, and environmental friendliness.^7–10^ A variety of materials have been extensively studied for adsorption. Traditional materials for extraction of uranium ions from aqueous solution, such as carbon,^11,12^ zeolite,^13^ clay,^14^ composites,^15^ natural polymers,^16^ and synthetic polymers,^17^ have abundant pores or functional groups. Among them, the binding capacity of chitosan is higher than that of commercial adsorbents, it is cheap and rich in nature, so it is of great interest to researchers for remediation of metal ions.^18,19^ However, the raw material of particle chitosan is usually difficult to recycle after adsorption reaction. Hydrogel, one of the most widely used polymers, consists of a three-dimensional network of hydrophilic polymers which allows diffusion of solutes into the interior network, and can be prepared using a covalent or a non-covalent approach with different functional groups such as carboxylic acid, hydroxyl and amine groups.^18,19^ PAA hydrogel has a specific adsorption capacity based on the mutual attraction between carboxyl and metal cations, and its synthetic steps are relatively timely, convenient, and flexible.^20^ Compared with chitosan, PAA has a stable and water insoluble structure. Furthermore, the bulk gel prepared from acrylic monomer holds recycle performance, hence research into CS/PAA composites is desirable.

In this work, a PAA-chitosan interpenetrating network is fabricated for sorption of uranium in potable water. The pH, contact time, and initial concentration were assessed by batch adsorption tests. The adsorption performance and mechanism were investigated by kinetic and isotherm models. PAA/CS composites were reused five times to predict properties of sustainable utilization. The experimental results indicate that the PAA/CS hydrogel has much potential in disposal of sewage.

## 2. Experimental

### 2.1. Materials

Acrylic acid (AA) and methylene-bis-acrylamide (MBA) were purchased from Sinopharm Chemical Reagent Co., Ltd. Chitosan (CS) and sodium hydrogen sulfite (SHS) were provided by Xilong Chemical Co., Ltd. Ammonium persulfate (APS) was supplied by Shanghai SANGON Biological Engineering Co., Ltd, China. A uranium stock solution (1000 mg L^−1^) was prepared by adding UO_2_(NO_3_)_2_·6H_2_O to deionized water. All chemicals used in this study were of analytical grade.

### 2.2. Preparation of PAA/CS

The chitosan (2 g) was dissolved in a beaker of acetic acid solution with a mass fraction of 20% by stirring for about 20 min at room temperature, and about 0.20 g APS and 0.20 g SHS was added to the solution with slow stirring. Then the acrylic monomer (14 mL) and 0.48 g MBA were put into a beaker, with ultrasound for 10 minutes. One of the solutions was poured into the other after both solutes were well-distributed. After the hydrogel formed, the product was washed with deionized water several times. Finally, the obtained hydrogel was dried in a vacuum freeze-drying machine for 12 h and stored in a vacuum drying oven at 55 °C.

### 2.3. Instrumentation

FTIR (Fourier transform infrared spectroscopy) of the samples was analyzed using a FTIR spectrophotometer in the range of 4000–400 cm^−1^.

SEM (scanning electron microscope) analysis of PAA/CS and PAA/CS after adsorption were recorded using Nova Nano 230 apparatus.

EDX (energy dispersive X-ray) measurements were carried out employing a scanning electron microscope as mentioned before.

### 2.4. Adsorption experiments

Batch adsorption experiments were performed by adding 0.01 g PAA/CS to a conical flask with 10 mL standard solution, of concentration 100 mg L^−1^ diluted by the stock solution and shaking at 150 rpm, 28 °C. After that, the concentration of supernatant in the system was analyzed by UV-vis spectrophotometer with a wavelength of 650 nm using Arsenazo-III as the complexing agent.

The U(vi) ion removal amounts on PAA/CS hydrogel were calculated as follows:1
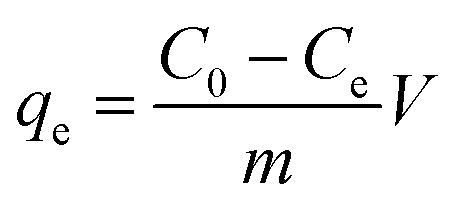
where *q*_e_ is the adsorption capacity of the PAA/CS (mg g^−1^), *C*_0_ is the initial concentration of U(vi) ions before adsorption (mg L^−1^), *C*_e_ is the final concentration of U(vi) ions after adsorption (mg L^−1^), *V* is the volume of U(vi) ion solution (mL), and *m* is the absorber mass supplied (g).

To investigate the effect of pH on adsorption, the pH value of solution before adsorption was adjusted to 1–5.5 with the diluted HNO_3_ (0.1 M) or NaOH (0.1 M) at initial U(vi) ions of 50, 100, and 150 mg L^−1^.

To assess the effect of initial ion concentrations on adsorption, the PAA/CS of 0.01 g was added to several flasks containing 10 mL solutions in which the initial U(vi) ions concentrations were 20–500 mg L^−1^ and pH 4.0 at 28 °C.

The isotherms were studied by adding 0.01 g PAA/CS in eight equal volume flasks containing 10 mL U(vi) ion solution with initial ion concentrations of 20, 50, 100, 150, 200, 300, 400, and 500 mg L^−1^, at 28 °C, with shaking for 300 min. The effect of contact time and the adsorption kinetic experiments were conducted using initial uranium concentrations of 50, 100, and 150 mg L^−1^, at 28 °C, with shaking for 350 min. Finally, the supernatants were taken at different time intervals (10, 20, 35, 50, 80, 110, 170, 230, 290, 350 min) and the residual U(vi) ion concentrations in solution were calculated.

After the adsorption reached equilibrium, the absorbent was soaked in elution solution (0.1 M HNO_3_) for about 12 h at room temperature and washed with distilled water to neutral. The regenerative adsorbent was reused five times.

## 3. Results and discussion

### 3.1. Characterization of PAA/CS

**Fig. 1:**
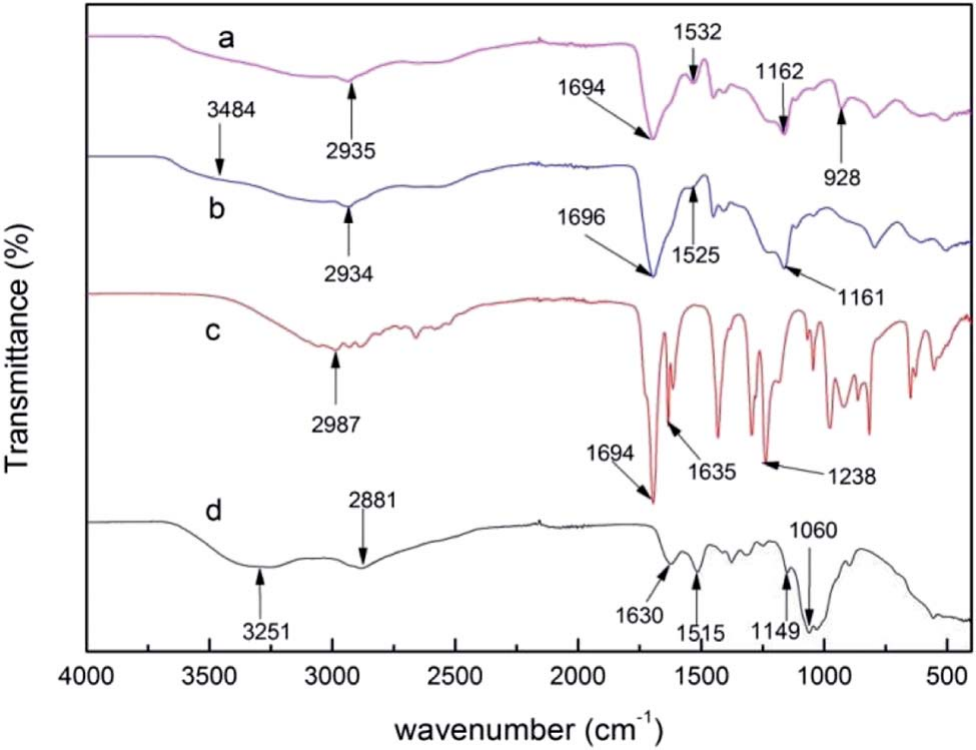
Fourier transform-infrared (FT-IR) spectra of (a) U-PAA/CS, (b) PAA/CS, (c) AA and (d) CS.

The infrared spectra of AA, CS, PAA/CS, and PAA/CS after adsorption in the region of 400–4000 cm^−1^ are depicted in Fig. 1. Strong absorption peaks appeared at 1694 cm^−1^, which indicated the stretching of the carboxylic group from AA, PAA/CS, and U-PAA/CS complexes. CS shows characteristic peaks at 3251, 1630, 1515, and 1060 cm^−1^ corresponding to the stretching of O–H, amide-I, amide-II, and the stretching of –C–O–C, respectively.^21,22^ The peak that disappeared at 1635 cm^−1^ from –C=C– is attributed to polymerization of AA monomer, and the emerging peaks at 1525 cm^−1^ (amide characteristic absorption bands) are assigned to the addition of CS, confirming the PAA/CS product formed. After adsorption, there was a new peak at 928 cm^−1^ from the stretching vibration of O=U=O, suggesting that uranium ions can be absorbed by PAA/CS composite.

**Fig. 2:**
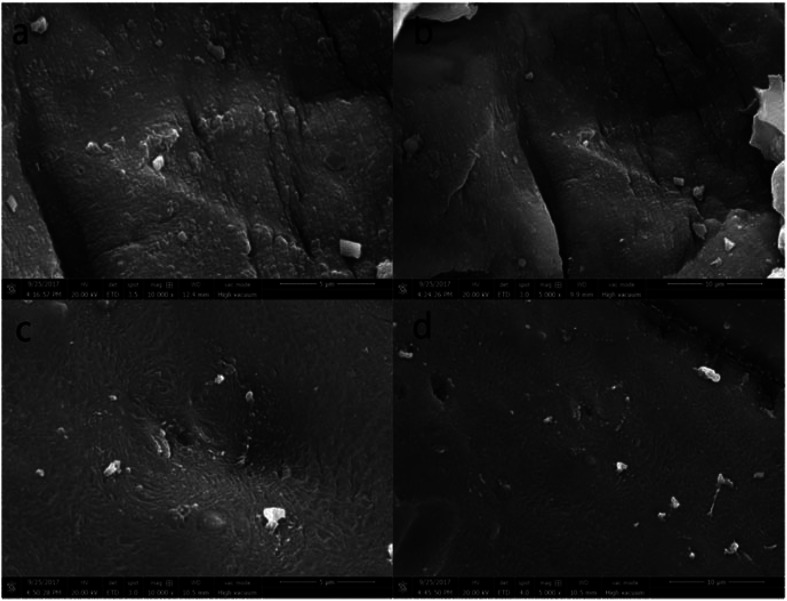
SEM images of PAA/CS. (a) and (b) Before adsorption, (c) and (d) after adsorption.

Scanning electron microscopy (SEM) images of PAA/CS and U-PAA/CS are shown in Fig. 2. As depicted in Fig. 2(c) and (d), the overall frame structure of composite was not changed after adsorption, which suggested that the gel after adsorption of uranium was no trail of destruction. The surface of the materials became denser and roughened during the diffusion of UO_2_^2+^ in many pores of hydrogels. The surface stripe of the adsorbent changed to a spiral, which could be attributed to the swelling of the uranium solution into the interior of the absorbent. The results showed the PAA/CS hydrogels had a strong binding capacity with UO_2_^2+^.

**Fig. 3:**
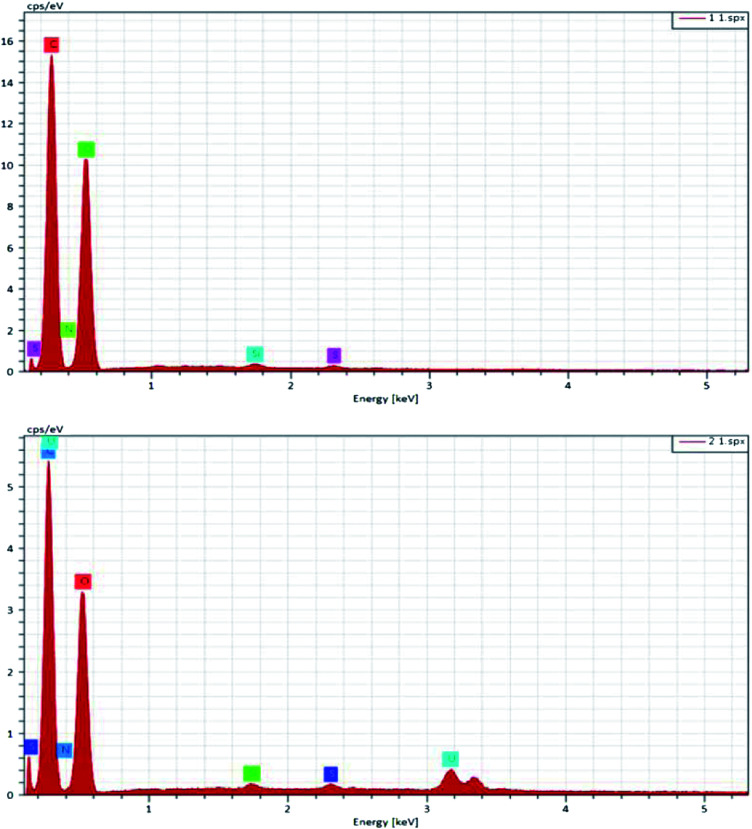
EDX spectra of PAA/CS. Before adsorption and after adsorption.

EDX analysis was conducted, and is depicted in Fig. 3. It is worth noting that the absorbent contains three elements, C, O, and N (Table 1), and after adherence of uranium onto the surface of PAA/CS, a new peak of U appeared in the EDX spectrum, confirming that the UO_2_^2+^ is successfully captured by PAA/CS.

**Table 1:** Elemental composition of the PAA/CS and U-PAA/CS by EDX

Samples	C (%)	N (%)	O (%)	U (%)
PAA/CS	54.66	5.60	39.56	0
U-PAA/CS	51.28	4.36	43.39	0.97

#### 3.1.1 Effect of initial concentration of the U(iv) ions

**Fig. 4:**
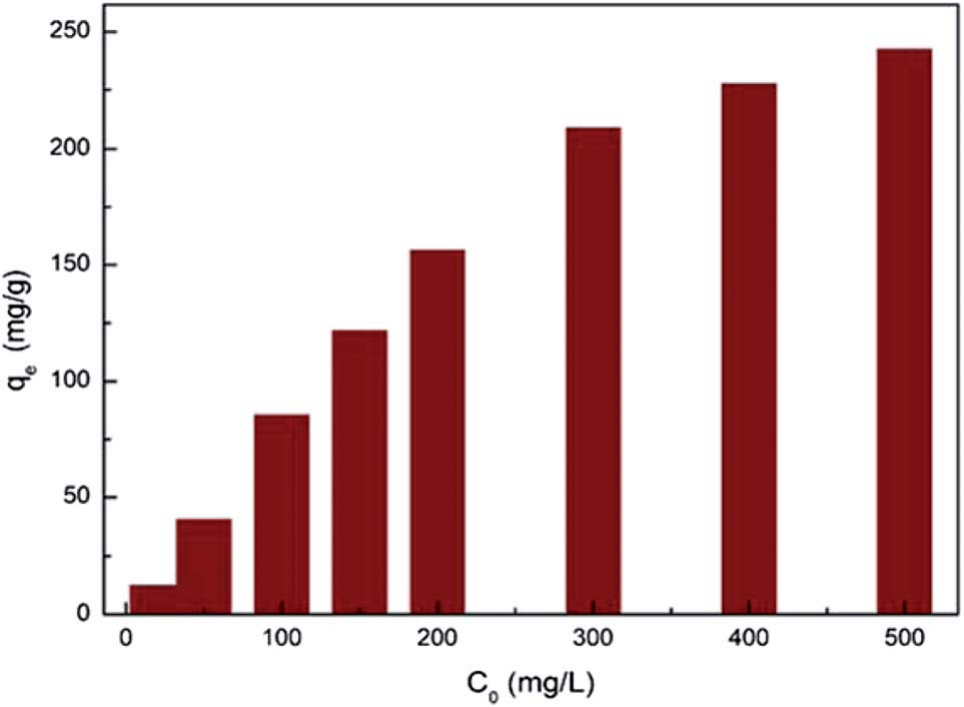
Effect of initial concentrations (20, 50, 100, 150, 200, 300, 400, 500 mg L^−1^) on U(vi) removal by PAA/CS hydrogels. (Volume, 10 mL; absorbent dose, 0.01 g; pH value, 4.0; temperature, 28 °C; rotating speed, 150 rpm).

The effects of initial U(iv) ions concentrations on the adsorption process by PAA/CS at 28 °C with pH = 4 are presented in Fig. 4, in which it can be seen that adsorption of U(iv) on PAA/CS increased with increasing concentrations of the U(vi) ions. This may be because the increase of concentration decreases mass transfer resistance between the solid and liquid phases.^23^ At the same time, with the concentration increased, the particular number of active binding sites determined that adsorption no longer occurred.

#### 3.1.2 Effect of initial pH

**Fig. 5:**
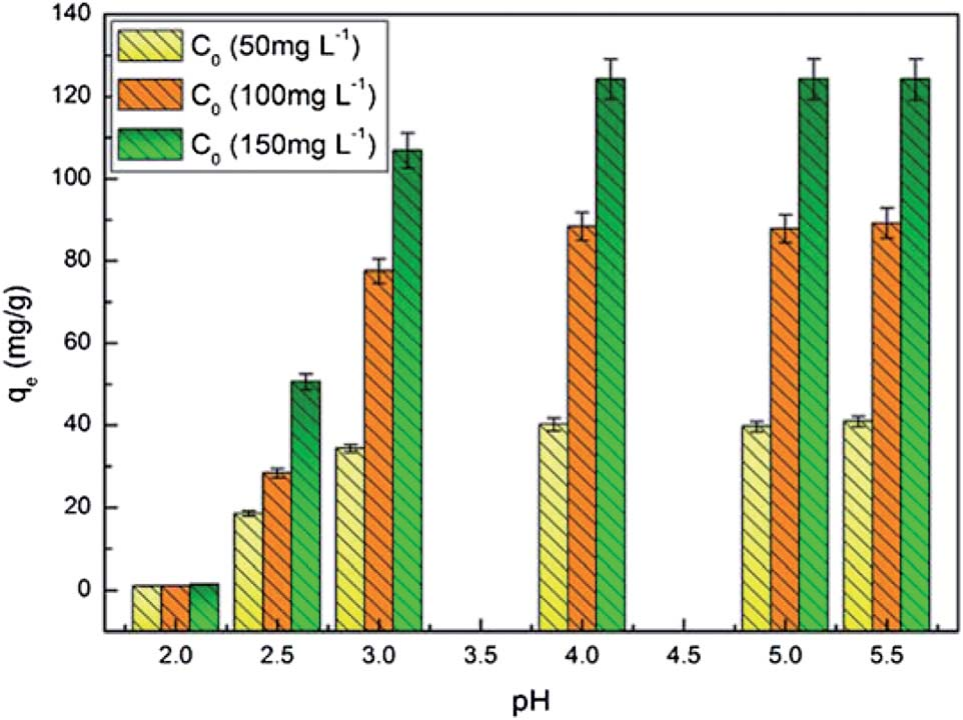
Effect of contact pH on U(vi) adsorption. (Volume, 10 mL; absorbent dose, 0.01 g; initial concentration, 50, 100, and 150 mg L^−1^; pH value, 4.0; temperature, 28 °C; rotating speed, 150 rpm).

The pH of aqueous solution affects the adsorption process as it can influence interaction between different ions in the system, and the adsorption of UO_2_^2+^ ions depends on distribution of the uranium species in solution.^24^Fig. 5 shows that the adsorption capacities of PAA/CS enhanced sharply because of deprotonation of the amino groups and carboxyl group with increase of pH from 2 to 4, and then the adsorption capacity showed independent on further increasing of pH and reached a plateau at pH 4, attributed to the fixed amount of –COO^−^, –OH, and –NH_2_ on absorber in aqueous solution. The decreased concentration of uranium in the aqueous phase suggests that U(iv) ions are adsorbed through ion-exchange with hydrogen ions on the binding sites formed by amino groups and through electrostatic attraction with carboxyl groups on PAA/CS hydrogel.

#### 3.1.3 Effect of contact time and kinetic studies

**Fig. 6:**
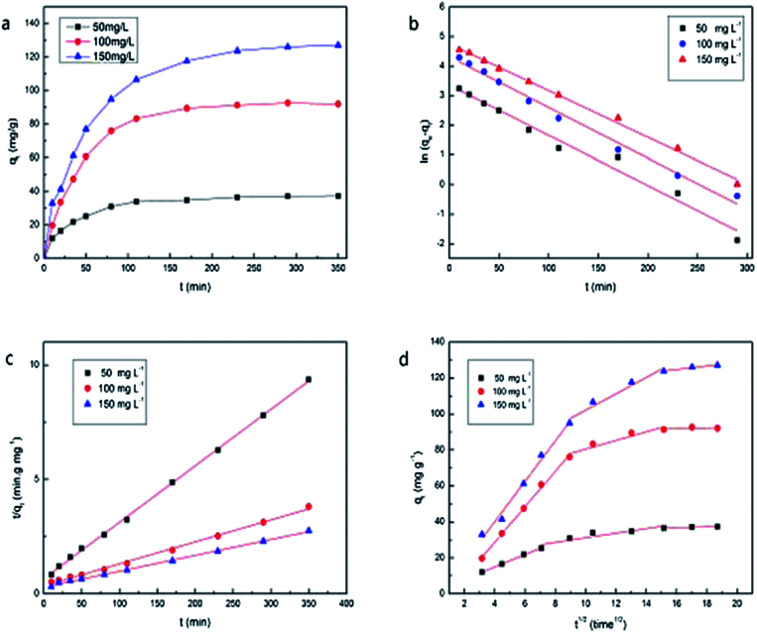
(a) Effect of contact time on U(vi) adsorption; (b) pseudo-first-order and (c) pseudo-second-order sorption kinetics; (c) intraparticle diffusion kinetics of U(vi) onto PAA/CS at various initial concentrations.

Fig. 6(a) shows the trend of adsorption *versus* contact time by PAA/CS. The rate of adsorption at the early stage was faster than that at the latter period, and the maximum adsorption capacity was obtained at the equilibrium stage (250 min). To allow most of the binding sites to be occupied by uranium ions and the adsorption end-point to be at a stable stage, 350 min was selected as the reaction time in subsequent experiments.

Kinetic studies revealed the dynamic process and the mechanism of sorption. Pseudo-first-order, pseudo-second-order kinetic models and intra-particle diffusion model are mainly used to fit the experimental data.

The pseudo-first-order model is expressed in the following equation:

2 ln(*q*_e_ − *q*_t_) = ln *q*_e_ − *k*_1_*t*

The pseudo-second-order model is expressed in the following equation:3
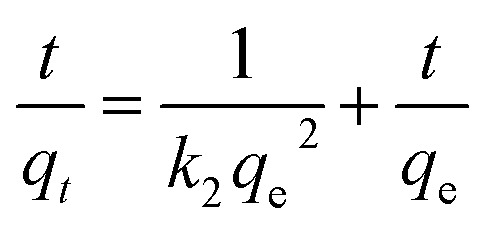
where *q*_e_ and *q*_*t*_ represent the absorbed amounts on PAA/CS at equilibrium and time *t* (min), respectively. *k*_1_ and *k*_2_ (min^−1^) are the rate constant of the pseudo-first-order model and second-order kinetic equation. *t* is the contact time of the adsorption process.

In addition, the model of intra-particle diffusion can be expressed as:

4 *q*_*t*_ = *k*_i_*t*^1/2^ + *C*

where *k*_i_ (mg g^−1^ min^−1/2^) is the rate constant of intra-particle diffusion, and *C* is thickness of the boundary layer.

As shown in Table 2, the *k*_1_, *k*_2_, and *q*_e_ values in each formula are calculated from the pseudo-first and the pseudo-second-order models, which showed that the pseudo-second-order model fits the experiment data better as the values of *q*_e,cal_ are closer to the experimental results (*q*_e,exp_) and the *R*^2^ of 0.977–0.996 obtained from the pseudo-second-order model were much higher than that calculated from the pseudo-first-order model.

**Table 2:** Pseudo-first-order, pseudo-second-order, and intra-particle diffusion kinetic model parameters

Kinetic model parameters	*T* = 301 K, pH = 4		
	50 mg L^−1^	100 mg L^−1^	150 mg L^−1^
**Pseudo-first-order model**			
*k*_1_ × 10^−2^	1.896	1.716	1.579
*R* ^2^	0.977	0.989	0.996
*q*_e_ (mg g^−1^)	28.6619	74.9686	115.8238

**Pseudo-second-order model**			
*k*_2_ × 10^−4^	9.285	3.943	1.661
*R* ^2^	0.999	0.996	0.998
*q*_e_ (mg g^−1^)	40.5186	85.4021	144.0922
*q*_e,exp_ (mg g^−1^)	37.369	82.731	127.162

**Intra-particle diffusion**			
*k*_1_	3.4362	9.8428	11.2239
*k*_2_	1.2724	2.4046	4.5495
*k*_3_	0.2118	0.2016	0.9545

The plots in Fig. 6(d) based on the intra-particle diffusion models provide an explanation of the adsorption process, which emphasizes that adsorption in aqueous solution can be viewed as three steps, instantaneous adsorption or external surface adsorption, diffusion into mesopores, and equilibrium stage. The rate of each step follows the order of *k*_1_ > *k*_2_ > *k*_3_.

#### 3.1.3 Adsorption isotherm studies

The adsorption isotherm is a model to investigate how the adsorbed ions are distributed over the adsorbent. From the research, Langmuir and Freundlich isotherm models are expressed as in eqn (5) and (6), respectively.5
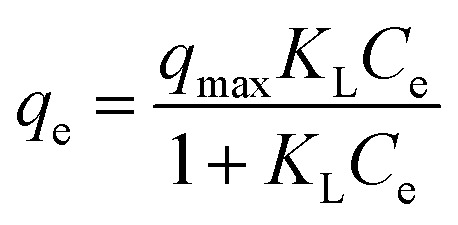

6 *q*_e_ = *K*_F_*C*^1/*n*^_e_

where *C*_e_ is the equilibrium concentration of the absorbate (mg L^−1^), *q*_e_ is the amount of absorbed ions at equilibrium (mg g^−1^). *q*_max_ represents the maximum adsorption capacity of the absorber (mg g^−1^). *K*_L_ and *K*_F_ are the Langmuir adsorption constant and Freundlich constant related to the energy of adsorption (L mg^−1^). *n* is the Freundlich constant related to the intensity.

The Temkin isotherm provides an assumption that the free energy of sorption is a function of surface coverage. The interaction of adsorbents and adsorbates has a significant effect on adsorption. The Temkin isotherm is written as:7
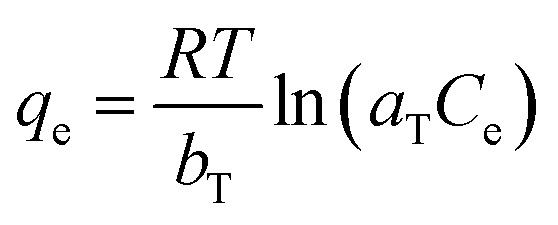
where *T* is the absolute temperature (K), *R* is the ideal gas constant (8.314 J mol^−1^ K^−1^), *a*_T_ is the equilibrium binding constant correlating to the maximum binding energy, and *b*_T_ is the Temkin isotherm constant.

The Langmuir isotherm model states that adsorption is a simple monolayer adsorption process on a homogeneous surface, while the Freundlich isotherm model predicts the forces between ions cannot be neglected and the surface of the absorbent is not homogeneous.

**Fig. 7:**
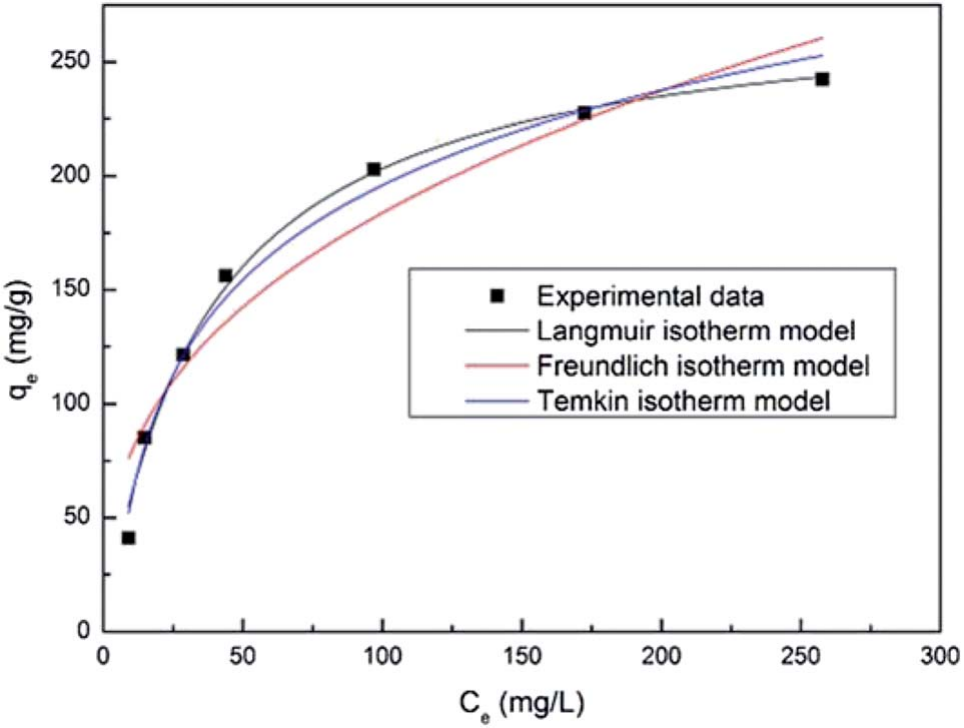
Langmuir, Freundlich, and Temkin isotherms for adsorption of U(vi) ions onto PAA/CS hydrogels. (Volume, 10 mL; absorbent dose, 0.01 g; initial concentration, 20, 50, 100, 150, 200, 300, 400 and 500 mg L^−1^; pH value, 4.0; temperature, 28 °C, rotating speed, 150 rpm).

Fig. 7 shows the fitting plot of the three isotherm models. As can be seen, the Langmuir isotherm model is found to be more in line with the experimental data, with the highest correlation value (*R*^2^ = 0.964) revealing a single layer adsorption process. The *K*_L_ value from the calculations of the Langmuir isotherm model is 0.02714 and the separation factor constant (0.0686 < *R*_L_ < 0.4243) suggests that the adsorption between the adsorbent (PAA/CS) and UO_2_^2+^ is favorable.

Based on the kinetic and isotherm studies, the mechanism of adsorption is probably a combination of the unpaired electrons of oxygen and nitrogen atoms with uranium ions through coordination bonds.^23^

#### 3.1.4 Recovery experiments and comparison with other adsorbents

**Fig. 8:**
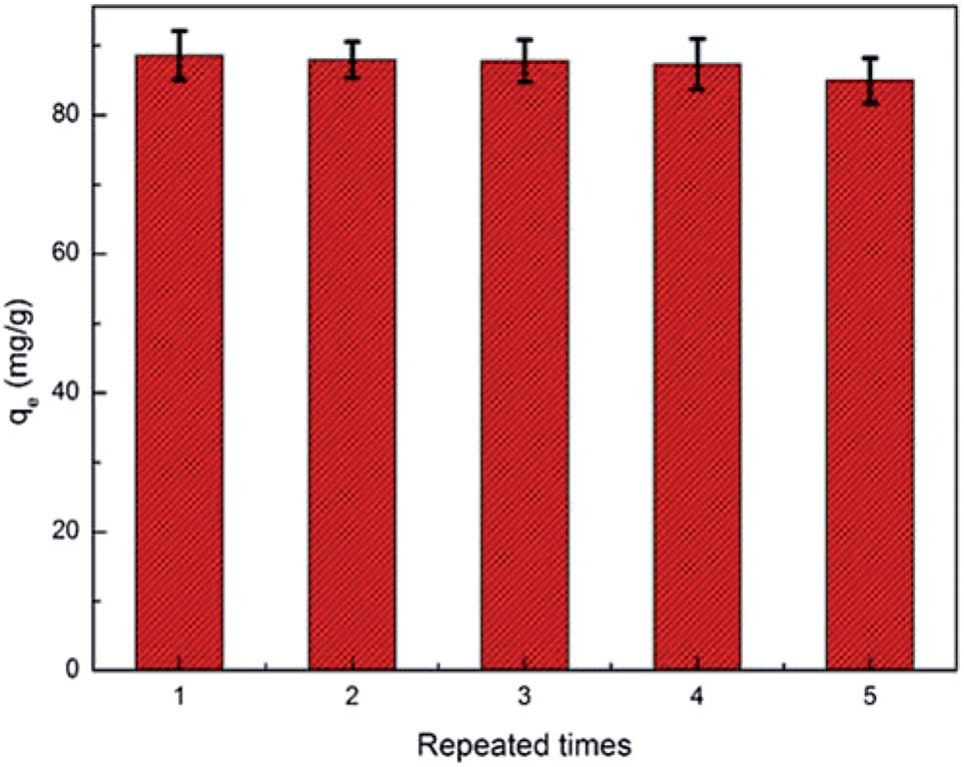
Adsorption/desorption cycles (initial concentration: 100 mg L^−1^).

The PAA/CS hydrogel was rinsed with 0.1 M HNO_3_ solution for investigating the reusable performance of adsorbent. According to Fig. 8, the capacity of composite in absorbing radioactive ions in water only slightly declined (by 4.04%). The outcome shows that the adsorbent in this study could be used at least five times.

As summarized in Table 3, comparison of organic, inorganic materials, and composites applied to extraction of contaminated ions in wastewater conducts that the PAA/CS hydrogel has more advantages including high efficiency of adsorption and simplicity of synthesis process than other adsorption materials.

**Table 3:** Comparison of adsorption capacities for U(vi) ion of various adsorbents

Absorbents	*Q*_max_ (mg g^−1^)	References
Zeolite	11.13	24
Oxidized activated carbon	25	25
Carboxymethyl cellulose (CMC)-grafted MWCNTs	112	26
Quinoline-8-ol-modified cross-linked chitosan	218	27
RGO	47	28
SA/CMC	101.8	29
AgOH–MWCNTs	140	30
PAA/CS	289.6	This work

## 4. Conclusions

A PAA/CS interpenetrating network hydrogel was prepared successfully in aqueous solution by free radical polymerization, and this hydrogel can be used in a simple and effective method for removal of the uranium from wastewater. The adsorption kinetics of PAA/CS were well described by a pseudo-second-order kinetic model. The experimental data fit the Langmuir model well and the maximum sorption capacity of PAA/CS was 289.6 mg g^−1^. Analysis of the hydrogel structure after adsorption gave the indication that PAA/CS composites have excellent prospects in management of polluted water.

## Conflicts of interest

There are no conflicts to declare.

## Supplementary Material
